# PARIN5, a Novel Thrombin Receptor Antagonist Modulates a Streptozotocin Mice Model for Diabetic Encephalopathy

**DOI:** 10.3390/ijms24032021

**Published:** 2023-01-19

**Authors:** Valery Golderman, Zehavit Goldberg, Shany Guly Gofrit, Amir Dori, Nicola Maggio, Joab Chapman, Ifat Sher, Ygal Rotenstreich, Efrat Shavit-Stein

**Affiliations:** 1 Department of Neurology, The Chaim Sheba Medical Center, Ramat Gan 52626202, Israel; 2 Department of Neurology and Neurosurgery, Sackler Faculty of Medicine, Tel Aviv University, Tel Aviv 6997801, Israel; 3 Goldschleger Eye Institute, The Sheba Medical Center, Ramat Gan 52626202, Israel; 4 Talpiot Medical Leadership Program, The Chaim Sheba Medical Center, Ramat Gan 52626202, Israel; 5 Sagol School of Neuroscience, Tel Aviv University, Tel Aviv 6997801, Israel; 6 Robert and Martha Harden Chair in Mental and Neurological Diseases, Sackler Faculty of Medicine, Tel Aviv University, Tel Aviv 6997801, Israel; 7 The TELEM Rubin Excellence in Biomedical Research Program, The Chaim Sheba Medical Center, Ramat Gan 52626202, Israel; 8 Department of Ophthalmology, Sackler Faculty of Medicine, Tel Aviv University, Tel Aviv 6997801, Israel

**Keywords:** diabetes mellitus, diabetic encephalopathy, thrombin, PAR1, NfL, TNF-α, behavioral tests

## Abstract

Diabetic encephalopathy (DE) is an inflammation-associated diabetes mellitus (DM) complication. Inflammation and coagulation are linked and are both potentially modulated by inhibiting the thrombin cellular protease-activated receptor 1 (PAR1). Our aim was to study whether coagulation pathway modulation affects DE. Diabetic C57BL/6 mice were treated with PARIN5, a novel PAR1 modulator. Behavioral changes in the open field and novel object recognition tests, serum neurofilament (NfL) levels and thrombin activity in central and peripheral nervous system tissue (CNS and PNS, respectively), brain mRNA expression of tumor necrosis factor α (TNF-α), Factor X (FX), prothrombin, and PAR1 were assessed. Subtle behavioral changes were detected in diabetic mice. These were accompanied by an increase in serum NfL, an increase in central and peripheral neural tissue thrombin activity, and TNF-α, FX, and prothrombin brain intrinsic mRNA expression. Systemic treatment with PARIN5 prevented the appearance of behavioral changes, normalized serum NfL and prevented the increase in peripheral but not central thrombin activity. PARIN5 treatment prevented the elevation of both TNF-α and FX but significantly elevated prothrombin expression. PARIN5 treatment prevents behavioral and neural damage in the DE model, suggesting it for future clinical research.

## 1. Introduction

Diabetes mellitus (DM) is a common disease with a global prevalence of 425 million patients [1]. DM causes peripheral nervous system (PNS) neuropathies [2] as well as central nervous system (CNS) damage. Diabetic encephalopathy includes mainly cognitive impairment and reduced mental speed and flexibility [3]. Pathological evaluation of DM encephalitic brains shows microvascular damage and neuronal loss [4].

Activation of the coagulation cascade and inflammation are tightly linked [5]. The cross-activation between the two is responsible for neuronal damage in a variety of diseases [6]. Specifically, in DM, hyperglycemia induces elevation of inflammatory factors, such as tumor necrosis factor α (TNF-α) and E-selectin in the CNS, and this is accompanied by blood–brain barrier (BBB) disruption and memory impairment [7]. Hyperglycemia is involved in molecular inflammatory mechanisms inducing TNF-α release and promoting apoptosis of hippocampal neurons. The coagulation system has been suggested to play a role in cognitive deterioration by several mechanisms, including damaging the endothelial microvascular cells. Thrombin plays a central role in glucose-induced endothelial injury, inducing TNF-α, interleukin 6 (IL6), matrix metalloproteinase 2 (MMP2), and MMP9, as well as oxidative stress proteins, by directly activating its cellular protease activating receptor 1 (PAR1) in brain endothelial cells. Glucose increases the activity of thrombin and the expression of TNF-α, IL6, MMP2, and MMP9, which are reduced by the thrombin inhibition [8]. The combined effect of the coagulation cascade protein thrombin and the inflammatory factor TNF-α further damages the endothelial barrier function [9], supporting a coagulation–inflammation interplay in the pathogenesis of diabetic encephalopathy. 

Thrombin mediates its cellular effects in the nervous system mainly by proteolytic activation of receptors and particularly PAR1, a G-protein coupled receptor [10]. PAR1 is also activated by other serine proteases such as FXa, plasmin, activated protein C (aPC), and MMPs [11,12,13,14]. PAR1 activation can cause either a neuroprotection [15] or a damage [16], depending upon the activation mode, activating cleavage site, and protease concentration. PAR1 pathway has been found to hold an important role in many neurological diseases, and its modulation has shown promising results in several models [17,18,19,20,21]. In the PNS, high levels of thrombin cause nerve conduction disruption mediated by PAR1 [17]. Thrombin activity is increased intrinsically in the sciatic nerve of the streptozotocin (STZ) diabetic-induced rat animal model and is associated with nerve conduction deficits and node of Ranvier (NOR) destruction. Specific thrombin inhibitors prevent these peripheral nerve conduction deficits and preserve the NOR structure [22]. In addition, the thrombin/PAR1/MMPs pathway is known to modulate neurotrophic levels and neuronal and glial damage [23]. Furthermore, thrombin, through PAR1 activation, interacts with brain pericytes to induce BBB dysfunction [24,25]. This data strengthens the importance of the thrombin/PAR1 pathway in neuromodulation.

The need for a specific thrombin-PAR1 modulator, which lacks significant anticoagulant properties and does not block PAR1 directly, enabling alternative protective activation, has prompted us to develop a class of compounds based on the thrombin binding site on PAR1, which include the PARIN5 molecule. PARIN5 includes a 5-amino-acid backbone blocked by a tosyl group on the N-terminus, which provides protection against degradation by amino peptidases. PARIN5 treatment in the STZ mouse model for DM improves peripheral nerve functions in measures of both nerve conduction and behavioral testing [19]. PARIN5 was developed as part of a group of molecules based on the structure of PAR1. A compound of this group was previously found to inhibit thrombin-induced PAR1 activation, as demonstrated in-vitro by the prevention of ERK phosphorylation [18]. Increased brain intrinsic thrombin activity has been found in several CNS pathologies [26,27,28,29,30] and is correlated with induced neuroinflammation. This has led us to study the involvement of the thrombin pathway in diabetic encephalopathy and specifically in the well-established STZ mice model. 

Sensitive measurement of serum neurofilament light chain (NfL) levels is rapidly gaining popularity as an accessible tool for evaluating neuronal damage [31,32]. NfL allows for the evaluation of neuronal damage in pre-clinical conditions as well [33], suggesting it as a possible screening test in many neurological diseases.

In the present study, we report behavioral changes in STZ diabetic mice, accompanied by the elevation of serum NfL levels as a highly sensitive marker for neuronal damage. Diabetic mice also developed elevated brain expression of TNF-α and FX, as well as changes in CNS and PNS thrombin activity. PARIN5 treatment normalized levels of serum NfL and TNF-α and FX expression, supporting a thrombin-PAR1-related mechanism in diabetic encephalopathy.

## 2. Results

### 2.1. Body Weight and Glucose Levels

STZ diabetic mice had significantly lower weights starting at day 11 following STZ induction (*p* = 0.007). The difference remained significant throughout the experiment. Treatment with PARIN5 did not affect the weights of the STZ diabetic mice. Glucose levels, as measured at the end of the experiment, did not differ between STZ diabetic mice and STZ diabetic mice treated with PARIN5 (575 ± 24, 535 ± 32.4 mg/dL, *p* = 0.4).

### 2.2. Serum NfL Levels

**Figure 1:**
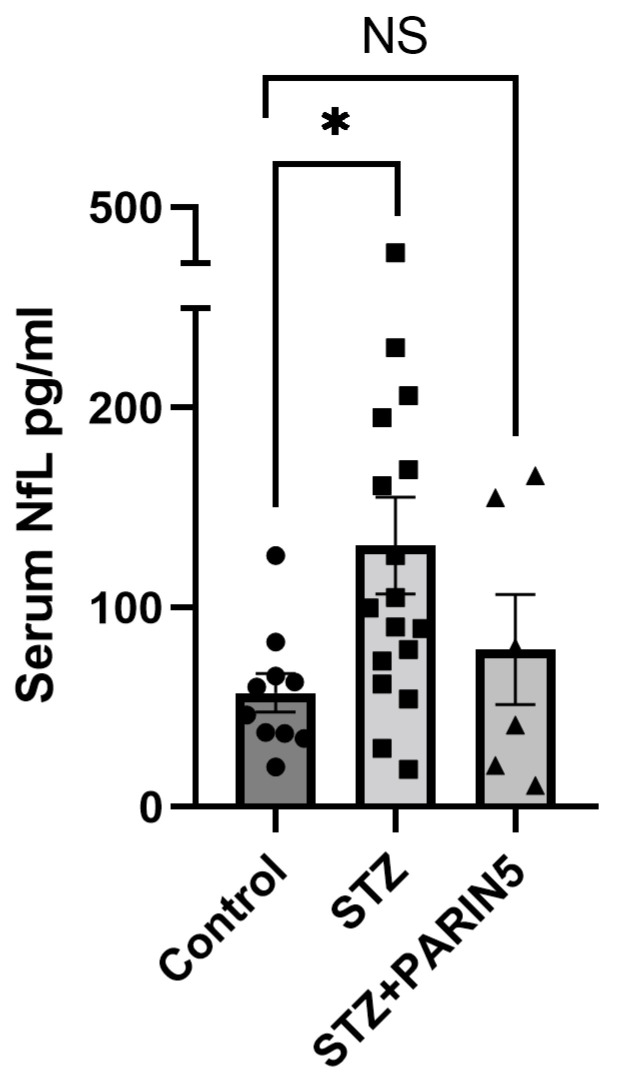
**Elevated levels of serum NfL in diabetic mice are normalized by PARIN5 treatment:** serum levels of NfL as a marker for neuronal damage are elevated in diabetic mice compared to the control. No significant (NS) change was measured in diabetic mice treated with PARIN5. Data are represented as mean ± SEM, * *p* < 0.05. Control = 10, STZ = 17, STZ + PARIN5 = 6.

Serum levels of NfL were significantly elevated in diabetic mice compared to control (131 ± 24.36, 57.23 ± 9.6 pg/mL, *p* = 0.035, Kruskal–Wallis with a post-hoc Dunn’s test, Figure 1). The elevated levels of serum NfL suggest axonal damage and neuronal cell death in diabetic mice. No significant difference was noted between control mice and diabetic mice treated with PARIN5 (79 ± 27.56 pg/mL, *p* > 0.99). The low levels of NfL in the PARIN5-treated group, although not significantly different compared to the diabetic untreated mice (*p* > 0.05), support a protective effect of PARIN5 against structural neuronal damage.

### 2.3. Thrombin Activity

**Figure 2:**
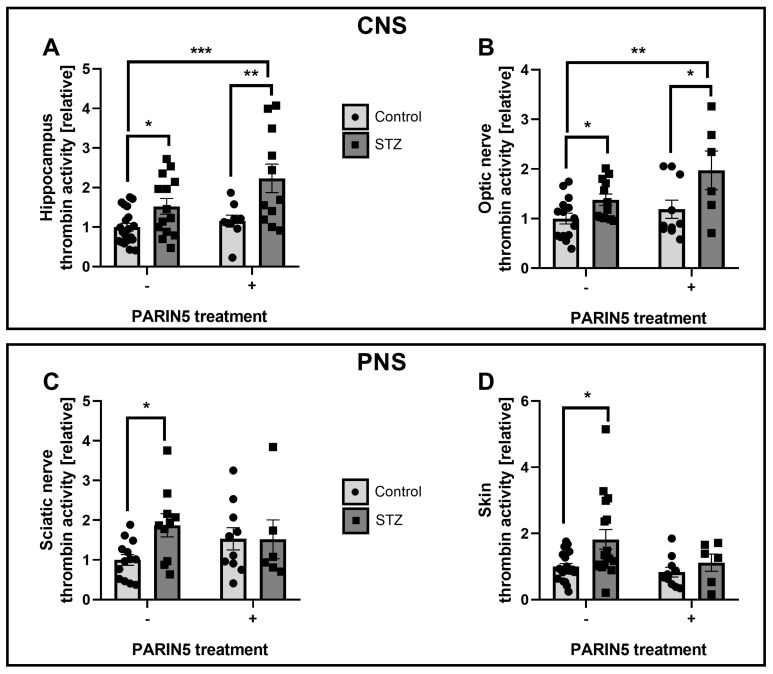
**Thrombin activity levels in the hippocampus, optic nerve, sciatic nerve, and skin:** Upper panel- CNS-derived tissues. (**A**) hippocampal thrombin activity was significantly elevated in STZ mice treated with PARIN5 compared to untreated controls and controls treated with PARIN5. (**B**) Optic nerve thrombin activity was significantly elevated in STZ with and without PARIN5 treatment compared to the control. Lower panel- PNS-derived tissues. Thrombin activity was significantly elevated in STZ compared to control in both sciatic nerve (**C**) and skin (**D**). Data are represented as mean ± SEM, * *p* < 0.04, ** *p* < 0.003, *** *p* < 0.0003. STZ = 10–17, STZ + PARIN5 = 6–11, PARIN = 9–10.

Thrombin activity was measured in tissues derived from STZ and control mice untreated and treated with PARIN5 (CNS: hippocampus, F (1, 50) = 0.7291; optic nerve, F (1, 39) = 0.1830. PNS: sciatic nerve, F (1, 35) = 3.009; skin, F (1, 52) = 0.6478). Thrombin activity measured in the CNS (hippocampus and optic nerve) was significantly elevated in the STZ diabetic mice compared to controls (hippocampus: 1.52 ± 0.15, 1 ± 0.09, *p* = 0.037, Figure 2A, optic nerve: 1.38 ± 0.1, 1.0 ± 0.1, *p* = 0.031, Figure 2B). Thrombin activity in the CNS was elevated in diabetic mice treated with PARIN5 compared to controls (hippocampus: 2.2 ± 0.36, *p* = 0.0003, Figure 2A. Optic nerve: 1.91 ± 0.36, *p* = 0.003, Figure 2B). We conducted a further experiment to study the exclusive effect of PARIN5 on thrombin activity in healthy mice. PARIN5 treatment in healthy mice did not change the measured thrombin activity. In addition, retinal observation revealed no edema, neovascularization, microaneurysm, or hemorrhage in STZ and PARIN5 treated mice, compared to control (by hematoxylin-eosin histology examination and spectral domain optical coherence, Figure S1). As expected, in the hippocampus and the optic nerve, treatment with PARIN5 in healthy control mice resulted in lower thrombin activity levels compared to STZ mice treated with PARIN5 (1.15 ± 0.15 and 1.18 ± 0.18, *p* = 0.008 and *p* = 0.03, respectively). In the PNS (sciatic nerve and skin), STZ mice had higher thrombin activity levels compared to controls (sciatic: 1.5 ± 0.48, 1.00 ± 0.13, *p* = 0.013, Figure 2C, skin: 1.1 ± 0.45, 1.00 ± 0.08, *p* = 0.02, Figure 2D). This increased activity was prevented by PARIN5 treatment (sciatic: 1.52 ± 0.48, *p* = 0.2, skin: 1.1 ± 0.25, *p* = 0.8). No direct effect of PARIN5 on thrombin activity in healthy mice was measured (sciatic nerve: 1.5 ± 0.3, *p* = 0.12, skin: 0.83 ± 0.14, *p* = 0.36). 

### 2.4. Behavioral Studies—Reduced Rearing Behavior on Open Field Test

**Figure 3:**
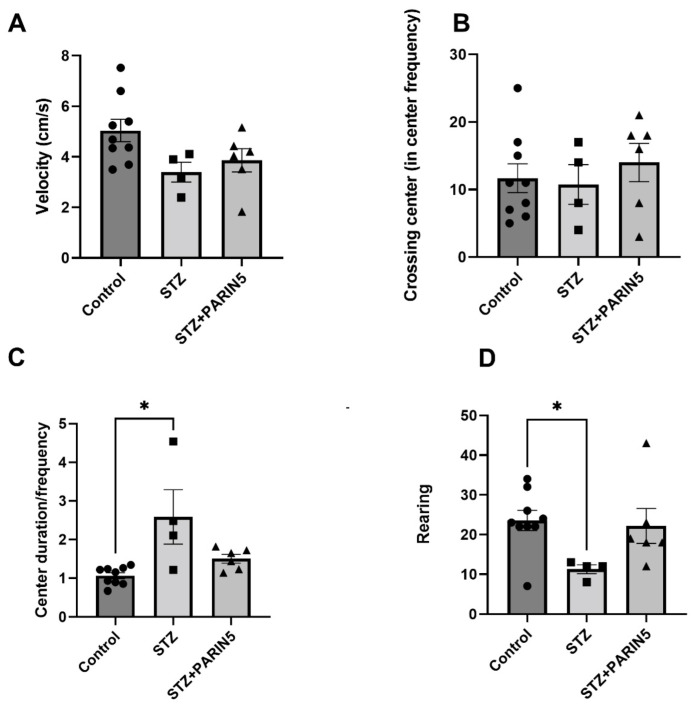
**Open Field test:** There were no significant differences between STZ diabetic and healthy mice in parameters of ambulation, including velocity (**A**) and frequency of center crossing (**B**). Center duration, divided by center frequency, was significantly longer in STZ diabetic mice compared to healthy controls, suggesting slower, more encephalopathic mice (**C**). Rearing behavior was significantly reduced in diabetic STZ mice compared to healthy control (**D**), supporting elevated anxiety as a possible manifestation of encephalopathy. Data are represented as mean ± SEM, * *p* < 0.05. Control = 9, STZ = 4, STZ + PARIN5 = 6.

STZ diabetic mice had a non-significant decrease in velocity compared to healthy controls (3.39 ± 0.39, 5.04 ± 0.44 cm/s, respectively, *p* = 0.06, Figure 3A), and similar frequency of crossing center (10.75 ± 2.9, 11.67 ± 2.1 respectively, *p* = 0.96, Figure 3B), supporting lack of significant motor deficit. There was no significant effect of PARIN5 treatment on these parameters. However, center duration normalized to center frequency showed a longer center duration of STZ diabetic mice compared to the control (2.58 ± 0.7, 1.06 ± 0.07, *p* = 0.02, Kruskal–Wallis with Dunn post-hoc analysis, Figure 3C). Rearing behavior, which indicates anxiety behavior in the open field, was significantly reduced in diabetic mice compared to controls (11.25 ± 1.1, 23.56 ± 2.55, respectively, *p* = 0.04, ANOVA with Dunnett’s post-hoc analysis, Figure 3D), supporting pathologically elevated anxiety as an encephalitic effect on the diabetic mice. PARIN5 treatment prevented this reduction in rearing since the difference in rearing was not significantly different in diabetic mice treated with PARIN5 compared to control (22.17 ± 4.4, 23.56 ± 2.55, *p* = 0.9).

### 2.5. Novel Object Recognition

**Figure 4:**
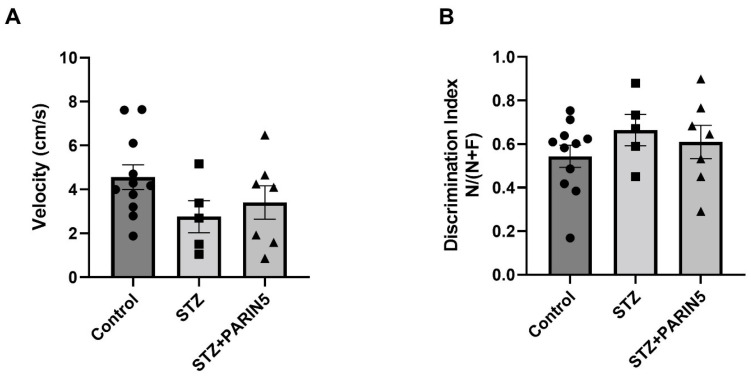
**Novel object recognition test:** there was no significant difference between healthy controls, STZ diabetic mice, and STZ diabetic mice treated with PARIN5 in either velocity (**A**) or discrimination index (**B**). Data are represented as mean ± SEM.

Results of velocity measured in the NOR test matched velocity results in the open field and did show a non-significant difference between the groups (4.69 ± 0.68, 2.16 ± 0.54, 2.89 ± 0.66 cm/s, for healthy controls, STZ diabetic mice, and STZ mice treated with PARIN5, respectively, *p* > 0.05, ANOVA, Figure 4A). The success in novel object recognition, as measured by recognition index, did not significantly differ between groups (0.55 ± 0.06, 0.66 ± 0.09, 0.6 ± 0.09, for healthy controls, STZ diabetic mice, and STZ mice treated with PARIN5, respectively, ANOVA, *p* > 0.5, Figure 4B).

### 2.6. Brain Gene Expression of TNF-α, FX, Prothrombin, and PAR1

**Figure 5:**
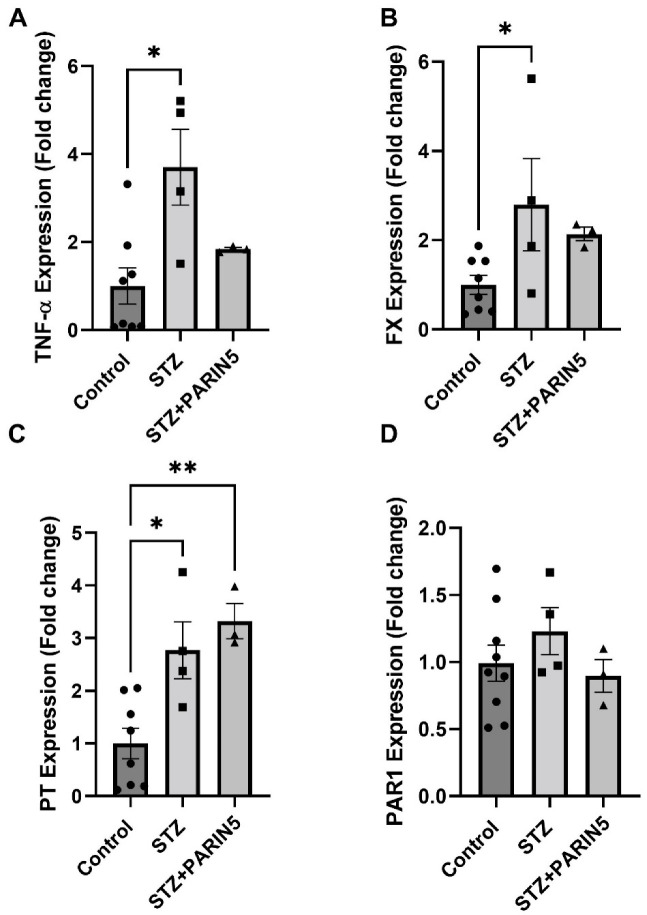
**Brain expression of inflammatory and coagulation factors following PARIN5 treatment:** expression of TNF-α (**A**) and FX (**B**) genes was significantly elevated in diabetic mice compared to control. Expression of prothrombin (PT) was significantly elevated in diabetic mice and was even higher in PARIN5-treated mice (**C**). Expression of PAR1 was not significantly affected by diabetes or PARIN5 treatment in diabetic mice (**D**). Data are represented as mean ± SEM, * *p* < 0.05, ** *p* < 0.01. Control = 8–9, STZ = 4, STZ + PARIN5 = 3.

Brain gene expression of TNF-α was significantly elevated in diabetic mice compared to controls (3.7 ± 0.86, 1 ± 0.4 fold change, *p* = 0.01, Figure 5A). PARIN5 treatment prevented this elevation, reducing the expression levels to levels similar to controls (1.83 ± 0.03 fold change, *p* = 0.5). Similar results were measured for FX expression, with significant elevation in diabetic mice compared to control (2.79 ± 1.93, 1 ± 0.2 fold change, *p* = 0.039, *t*-test, Figure 5B). Prothrombin (PT) was elevated in diabetic mice as well (3.3 ± 0.3, 1 ± 0.2 fold change, *p* = 0.01, Figure 5C); however, diabetic mice treated with PARIN5 had significantly higher PT expression compared to controls (3.3 ± 0.3 fold change, *p* = 0.004). No significant difference was measured in PAR1 expression (0.99 ± 0.13, 1.23 ± 0.17, 0.89 ± 9.1 fold change for control, diabetic, and diabetic treated with PARIN5, respectively, Figure 5D).

## 3. Discussion

In this study, we investigated the behavioral, inflammatory, and coagulation changes in the CNS in a diabetic mice model and evaluated the specific role of coagulation by pharmacologically modulating PAR1. 

We report a significant elevation in serum NfL, a subtle behavioral and cognitive deficit, an increased CNS intrinsic expression of coagulation and inflammation factors, and increased thrombin activity in diabetic mice. Together, these data support CNS structural damage in this STZ-diabetes-induced mouse model. Treatment with the thrombin receptor modulator was associated with NfL levels similar to controls. Though the difference between treated and untreated STZ mice did not reach significance, perhaps due to the limited sample size, these findings are potentially important in view of the significant elevation in NfL by STZ DM induction compared to controls. Increased serum NfL levels represent the existence of ongoing axonal damage and neuronal cell death. Serum NfL levels, as a marker for neuroinflammation-induced brain damage, were previously described in a number of diseases [34,35,36]. Recently, high levels of serum NfL were found to correlate with mild cognitive decline in patients with diabetes mellitus type 2 (DM2) without a prior significant insult, such as surgery. This elevation is reported at a very early stage of the disease, prior to recognition by more coarse diagnostic tools, such as the mini-mental state examination [37]. Our results in agree with these publications, supporting an elevation of serum NfL in the presence of subtle cognitive changes in the STZ diabetic mice. Peripheral neuropathies are also known to cause increased serum NfL [38,39]. The increased serum NfL levels observed in the present study may represent CNS damage, PNS damage, or a combination of the two. Though our results do not definitively distinguish the source of the serum NfL, it was found that extensive brain damage, such as in the experimental autoimmune encephalitis (EAE) animal model for multiple sclerosis [40], significantly increased serum NfL levels (over 100 fold increase). Also in more subtle brain damage, as can be found following status epilepticus [41] increases serum NfL levels were measured (1.5–2 fold). Behavioral changes in the diabetic mice, together with changes in brain gene expression, which were attenuated by PARIN5 treatment, support CNS involvement and origin for at least some of the serum NfL. The increased TNF-α mRNA expression in the brain, which was not found following the PARIN5 treatment, supports the involvement of the coagulation system in the inflammatory process taking place in the diabetic brain. 

The beneficial effect of PARIN5 on STZ diabetic animals is supported by a number of behavioral and biochemical measures and may involve an effect in a number of locations. Inflammation is related to blood–brain barrier (BBB) integrity and function [42]. Elevation of BBB permeability with tight junction damage and CNS elevation in the expression of TNF-α, IL-1β, and IL-6 in a diabetic STZ model, were previously described in conjunction with cognitive damage [43] and could be the target for PARIN5 beneficial effects. It is also possible that PARIN5 affects inflammation and/or coagulation in the periphery, which, in turn, leads to an indirect CNS effect. In the present study, thrombin activity in peripheral tissues (sciatic nerve and skin) was elevated in the STZ diabetic mice, in line with previously described effects [19]. Modulation of PAR1 activation by PARIN5 in diabetic mice resulted in lower peripheral thrombin activity, as expected. Indeed, in our previous studies, STZ-induced diabetes was found to cause nociceptive behavior in mice and rats indicated by hot-plate and rotarod tests; both were modulated by thrombin inhibitors [19,22]. Surprisingly, in the CNS (hippocampus, optic nerve), PARIN5 treatment in diabetic mice led to further elevated thrombin activity together with a greater elevation in prothrombin expression, perhaps supporting a feedback loop with upregulation of prothrombin production. A possible explanation for this apparent discrepancy is the source of thrombin, which may be glial and neuronal [44]. Another possibility is a temporal difference between the effect of PARIN5 in the periphery, causing a later indirect CNS effect. Thus, PARIN5 first reduces peripheral thrombin activity and later reduces CNS thrombin activity, a reduction which was not measured in the time point used in the present study. Alternatively, one may speculate that the increased thrombin activity measured in the PARIN5-treated animal’s brain is part of a complex feedback loop following excessive thrombin inhibition. Further experiments at different time points may clarify this point. Either way, PARIN5 modulates central nerve damage evidenced by decreased NfL, markers of inflammation, coagulation factors mRNA expression, and the prevention of behavioral changes. In addition, PARIN5 treatment seems to have a selective effect on pathologically induced thrombin activity rather than basal thrombin activity, which is a beneficial property of this compound. 

Diabetic cognitive impairment is characterized by slower data processing speed, memory impairment, difficulties in executive functions, attention, and mental flexibility [45]. In the present study, diabetic animals showed reduced rearing, which may correspond to elevated anxiety [46], and longer center duration to center entry frequency ratio in the open field test, supporting a cognitive slowing. These were present with no significant motor involvement, as supported by the speed of movement. These deficits were not found in treated STZ mice, emphasizing PARIN5’s beneficial CNS effects. 

Hyperglycemia is known to be related to CNS inflammation and to coagulation cascade activation [6,47]. In the present study, significant elevated TNF-α and FX expression levels were measured in the diabetic mice brain, and both were modulated by PARIN5 supporting CNS inflammation and coagulation interplay in diabetic encephalopathy. Hippocampal expression of prothrombin was elevated in diabetic mice, suggesting upregulation of intrinsic thrombin production in the CNS. This is in line with previous reports of elevated thrombin activity in inflammatory CNS conditions, such as MS and its animal model EAE [28,48], with an elevation of endogenous thrombin inhibition [49] and improvement of disease with exogenous thrombin inhibition [50]. In the present study, PAR1 expression in the brain was not significantly affected by diabetes or by PARIN5 treatment in diabetic mice.

It is interesting to note that PARIN5 did not affect either glucose levels or weight, implying that its beneficial effects are unrelated to glycemic control. Improvement of cognitive function and inflammation regardless of glucose levels described before [51] suggests a mechanism of diabetic encephalopathy that is far more complex than simple glucose toxicity and its metabolic associated effects and supports the need for developing specific pharmacological interventions in other neural-associated mechanisms.

Limitations of the study include characterization of PARIN5 effects and place of action. We did not directly measure BBB penetration of PARIN5 and its CNS levels. The timeline of our experiment was relatively short, with sacrifice and measurements at 43 days following STZ injection. Although NfL was previously demonstrated as an early marker for neuronal pathology, further studies, including the assessment of PARIN5 effects on NfL in even shorter and longer experiments, as well as the evaluation of thrombin activity dynamics at several time points, are needed. We began treating with PARIN5 at a very high glucose level, which may not reflect the clinical scenario, in which, hopefully, patients get diagnosed at earlier stages of their disease. Thus, perhaps a more significant cognitive improvement may be present with the beginning of treatment at lower glucose levels. This hypothesis will be evaluated in future research. Several reports indicate an association between MMPs, thrombin, and PAR1 and their effects on glial cells and BBB penetration in diabetes [23,24,25]. This strengthens the importance of this pathway in CNS-involved diabetic pathologies and suggests further research. Since PARIN5 is a PAR1 modulator, it is a potential anti-coagulant. We previously conducted PT PTT measurements of PARIN5 in human blood without a significant elevation [19], and in this current study, we did not detect any bleeding side effects in the treated animals. Furthermore, PARIN5-treated animals did not differ in general health compared to the controls. However, a specifically dedicated experiment for coagulation side effects should be conducted to allow the use of higher doses of PARIN5 treatment. 

The present results were obtained in a mouse model, and the relevance of PAR1 to DE treatment in human subjects remains to be verified in clinical studies. Measuring NfL levels in diabetic patients and correlating it with cognitive evaluation results is a topic for future research and may aid in the early diagnosis of diabetic encephalopathy. The primary outcome of the present study was the evaluation of the STZ diabetic model effect on parameters of neuronal damage, such as NfL levels and neuroinflammation involved in CNS and PNS coagulation activity levels. The STZ model used in the current study causes severe diabetes with an expected large effect size; thus, our sample size was calculated accordingly. Evaluating PARIN5 was a secondary outcome, and although PARIN5 treatment resulted in no significant difference between treated animals and healthy control in many of the evaluated parameters, it was of borderline significance when comparing PARIN5 treated to untreated STZ diabetic mice. Evaluating PARIN5 effects, as well as dosage range, treatment protocols, and administration, is the subject of future studies. Further characterization of the PARIN5 effect in early vs. late intervention in diabetic encephalopathy and as a potential preventive treatment is an additional important step toward drug development. An evaluation of PARIN5 CNS penetrance and presence is also needed and will shed light on the mechanism of this promising treatment. 

## 4. Materials and Methods

### 4.1. Animals

Male C57BL/6, 8 and 12-week-old mice (Envigo, Jerusalem, Israel) were maintained in a controlled facility at 18–22 °C, 40–60% humidity, with 12 h dark/12 h light cycles, and allowed free access to water and food. All animal procedures and experiments were approved by the Sheba Medical Center Institutional Animal Care Committee (920/14/ANIM) and conformed to recommendations of the Association for Research in Vision and Ophthalmology Statement for the Use of Animals in Ophthalmic and Vision Research and according to the ARRIVE (Animal Research: Reporting of In Vivo Experiments) guidelines.

### 4.2. Diabetes Induction

All experiments were initiated at least one week following the arrival of the mice to the institutional animal facility to attenuate stress. Diabetes was induced by a single (150 mg/kg) intraperitoneal (IP) injection of streptozotocin (STZ, S0130, Sigma-Aldrich, St. Louis, MO, USA, 0.1 M sodium citrate buffer, pH 4.5), as previously described [19,52]. Glucose levels were measured by a commercial glucometer device (Xpress-i, Nova Biomedical) on three repeated occasions within a week from STZ injection, at a constant time during the day, with no prior fasting. Mice were considered hyperglycemic when glucose levels were above 250 mg/dL.

### 4.3. Treatment Protocol

PARIN5 molecule was synthesized by American Peptide Company (Sunnyvale, CA, USA). The PARIN5 molecule included a 5-amino-acid backbone and was designed based on the thrombin recognition site sequence of the PAR1 N-terminal (^35^NATLDPR^41^). The length of the backbone amino acids was tested before in a preliminary study and was chosen according to its high efficacy and its safety profile. This family of compounds was previously evaluated in a glioblastoma model and reduced PAR1 activation by thrombin, as evidenced by a reduced ERK phosphorylation [18]. The molecule is protected by a tosyl group at the N-terminal and conjugated to chloro-methyl-ketone (CMK) at the C-terminus, which serves as a serine active site blocker. The purity grade is over 96% which was verified by HPLC.

The experiment was repeated four times: The first three repetitions were carried out on eight-week-old mice and included three groups: (1) control—healthy mice, (2) STZ—diabetic mice, (3) STZ + PARIN5—diabetic mice treated with PARIN5 dissolved in saline used as a vehicle (375 ng/kg, 100 nM). Experiment #1: Control (n = 8), STZ (n = 4), STZ + PARIN5 (n = 3). This cohort was used to assess for gene expression analysis. Experiment #2: Control (n = 11), STZ (n = 7), STZ + PARIN5 (n = 9). This cohort was used for behavioral analysis. According to human endpoints, four animals were sacrificed. Following Outlier identifier analysis, another two animals were excluded; therefore, the final numbers of the groups were 10, 4, and 6, respectively. Experiment #3: Control (n = 4–14), STZ (n = 10–17), STZ + PARIN5 (n = 5–11). This cohort was used for assessing thrombin activity and serum NfL levels. 

The fourth repetition (experiment #4) was carried out on 12-week-old mice and included two groups: (1) control-healthy mice (n = 9), (2) PARIN5-treated healthy mice (n = 9–10), and was used for measuring thrombin activity and serum NfL levels. 

**Figure 6:**
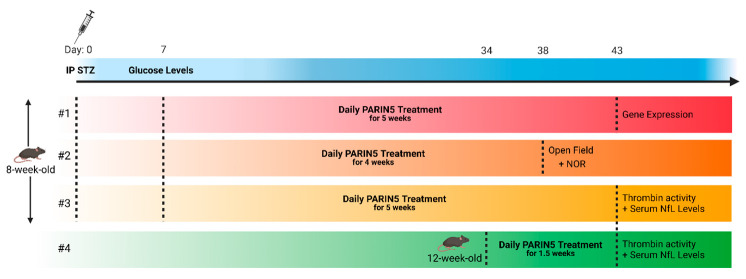
Experiment timeline. Created with BioRender.com.

PARIN5 and control saline treatments were administered by IP injection. Experiments #1–3: once daily for four to five weeks, starting one week following STZ injection. Experiment #4: once daily for 1.5 weeks (Figure 6).

### 4.4. Neurofilament Light Chain (NfL)

Blood samples collected following decapitation were kept at room temperature for 30 min and then centrifuged for 10 min at 1500× *g*. The concentration of serum NfL was measured using the Simoa NfL assay (Quanterix, Quanterix Corp, Boston, MA, USA) NF-light Advantage kit (UmanDiagnostics, Umea, Sweden). The Simoa instrument uses bead-conjugated immunocomplex, which was applied to a multi-well array. The average number of enzymes per bead (AEB) was used for calibration by measurements on bovine NfL (UmanDiagnostics) serially diluted in assay diluent. 

### 4.5. Thrombin Activity Assay

Thrombin activity was measured by a fluorescence emitted by the cleavage of Boc-Asp (OBzl)-ProArg-AMC (I-1560; Bachem, Bubendorf, Switzerland) as previously described [53]. Following mice sacrifice by phenobarbital injection, hippocampi were removed and placed in 96 well black plates (237108, Nunc, Roskilde, Denmark) containing buffer (in mM: 50 TRIS/HCl pH 8.0, 150 NaCl, 1 CaCl_2_, and 0.1% BSA). The substrate containing aminopeptidase and prolyl endopeptidase inhibitors (bestatin, 70520, Cayman Chemical Company, Ann Arbor, MI, USA, 0.1 mg/mL, and prolyl endopeptidase inhibitor, 537011, Merck, Darmstadt, Germany, 0.2 mM) at a volume of 14 μM was added to the wells. Fluorescence emitted was measured by the microplate reader (Tecan; Infinite F Nano+; Männedorf, Switzerland). Bovine thrombin (Sigma, T4648) was used for calibration.

### 4.6. Behavioral Studies

The experiments were always conducted at the same time of day to avoid the circadian cycle effect (between 9 am to 15 pm). All behavioral tasks were performed in polymethyl meth-acrylate apparatus. The arenas were cleaned and dried between each session. The tasks were performed in a room dedicated to long-term behavioral procedures and conducted by the same experimenter who was blinded to the animal group allocation. During the behavioral experiments, all efforts were made to minimize interruptions, such as door openings and the sights and smells of people. During the same behavioral tasks, the apparatus-surrounding area remained unchanged to avoid un-balanced cues in spatial memory tasks.

#### 4.6.1. Open Field

As previously described [54], mice were placed in the middle of the open field apparatus consisting of a square box (47 × 47 × 29 cm), and each mouse was recorded for five min (to avoid noise disturbance, the recording began with a delay of 30 s from the moment the mouse was put in the center of the arena). To remove olfactory cues, the floor and walls of the field were thoroughly cleaned with ethanol and air-dried after each trial. Each trial was recorded with a ceiling-mounted video camera (Tracker VP200; HVS Image, Hampton, UK) for later analysis. The data analysis was performed by a tracking system (EthoVision XT by Noldus, Wageningen, The Netherlands). Data analysis was conducted by a researcher blinded to the experimental group and included measurements of velocity, center frequency, center duration divided by center frequency as a measure of anxiety reduction, and rearing behavior as another measure of anxiety [46]. 

#### 4.6.2. Novel Object Recognition 

Recognition memory was assessed by the novel object recognition test (NOR), as previously described [55]. An open field arena measuring 47 × 47 × 29 cm was used for the experiments. Animals were placed in the arena for 5 min 24 h prior to experiments for habituation. For the acquisition phase, two heavy objects were placed in a symmetrical position in the arena. For the discrimination phase, which was performed 24 h following the acquisition phase, one of the objects was replaced by a novel one and exploratory behavior was assessed for 5 min. The arena was cleaned with 70% ethanol to prevent olfactory ques. 

Data acquisition and analysis were performed by EthoVision XT (Noldus, Wageningen, The Netherlands), and the behavior was quantified by the object nose touching at a distance less than 2 cm and/or nose touching [56]. Discrimination of visual novelty was assessed by a discrimination index defined as

(1) $$\frac{\mathrm{The}\;\mathrm{exploration}\;\mathrm{time}\;\mathrm{devoted}\;\mathrm{to}\;\mathrm{the}\;\mathrm{novel}\;\mathrm{object}—\mathrm{the}\;\mathrm{time}\;\mathrm{devoted}\;\mathrm{to}\;\mathrm{the}\;\mathrm{familiar}\;\mathrm{object}}{\mathrm{The}\;\mathrm{total}\;\mathrm{amount}\;\mathrm{of}\;\mathrm{exploration}\;\mathrm{of}\;\mathrm{the}\;\mathrm{novel}\;+\;\mathrm{familiar}\;\mathrm{objects}}$$

### 4.7. Real-Time Polymerase Chain Reaction (RT-PCR)

A half brain was placed in 1 mL Trizol reagent (15596026, Thermo Fisher, Waltham, MA, USA) and homogenized with a bullet blender homogenizer (Next Advance, Troy, NY, USA) at a maximum speed for 1 min. After solubilization, 200 µL chloroform was added to cause phase separation, where proteins are extracted to the organic phase, DNA resolves at the interface, and RNA remains in the aqueous phase. The consequent RNA phase cleaning was performed using Bio-Rad Aurum (732-6820, Bio-Rad Laboratories, Hercules, CA, USA). RNA samples purity, *A*_260_/*A*_280_ > 2.00, was considered acceptable for further analysis [57] (Supplementary Table S1). One microgram of total RNA was used for reverse transcription using a high-capacity cDNA reverse transcription kit (N8080119, Applied Biosystems, Rhenium, Israel). The quantitative real-time polymerase chain reaction was performed on the StepOne™ Real-Time PCR System (Applied Biosystems) using Fast SYBR Green Master (ROX) (4385612, Applied Biosystems). Hypoxanthine guanine phosphoribosyltransferase (HPRT) served as a reference gene in this analysis. A standard amplification program was used (1 cycle of 95 °C for 20 s, 40 cycles of 95 °C for 3 s, and 60 °C for 30 s). The primers used in this analysis are listed in Table 1. The results were normalized to reference gene expression within the same cDNA sample and calculated using the ΔCt method. Results are reported as fold changes relative to the control brains of sham animals and reported as mean ± SEM.

### 4.8. Statistics

The sample size was calculated using G-power, 3.1.9.7, with the following parameters: effect size—0.9, α type error—0.05, power—0.8. Statistical analyses and graphs were conducted using GraphPad Prism (version 7.00 for Windows, GraphPad Software, La Jolla, CA, USA). Prior to the final analyses, “Identify Outlier” analyses were conducted on all data sets. Identified outliers were omitted from all measures in the same analysis. Paired *t*-tests, one-way ANOVA, and two-way ANOVA, followed by a post-hoc test, were applied to normally distributed data sets. One-way ANOVA was followed by either Dunnett’s or Tukey’s post-hoc analyses. The normality of data was evaluated using the Anderson–Darling test. The Mann–Whitney test was applied to non-normal distributed data sets. Grouped, non-normal distributed data sets were transformed prior to analysis and evaluated by two-way ANOVA followed by the two-stage step-up method of Benjamini, Krieger, and Yekutieli. Results are expressed as mean ±SEM; *p* values < 0.05 were considered significant.

## 5. Patents

PAR-1 BASED THERAPEUTIC CONJUGATES AND USES THEREOF International publication number: WO15173802, Granted US patent US10028999.

## Figures and Tables

**Table 1:** Primers.

Gene	Forward	Reverse
HPRT	GATTAGCGATGATGAACCAGGTT	CCTCCCATCTCCTTCATGACA
PAR1	GCCTCCATCATGCTCATGAC	AAAGCAGACGATGAAGATGCA
FX	GTGGCCGGGAATGCAA	AACCCTTCATTGTCTTCGTTAATGA
TNF-α	AGATCAATCGGCCCGACTATCTC	GTTTGGGAAGGTTGGATGTTCGT
PT (prothrombin)	CCGAAAGGGCAACCTAGAGC	GGCCCAGAACACGTCTGTG

## Data Availability

Data is available from the corresponding author upon request.

## Supplementary Materials

The following supporting information can be downloaded at: https://www.mdpi.com/article/10.3390/ijms24032021/s1.
