# Supplementary material for: PARIN5, a Novel Thrombin Receptor Antagonist Modulates a Streptozotocin Mice Model for Diabetic Encephalopathy
Source: Int J Mol Sci. 2023 Jan 19;24(3):2021. doi: 10.3390/ijms24032021 (PMC9917200; doi:10.3390/ijms24032021)
Supplement: Supplementary file 1 [file ijms-24-02021-s001.zip › ijms-2158835-supplementary.pdf]

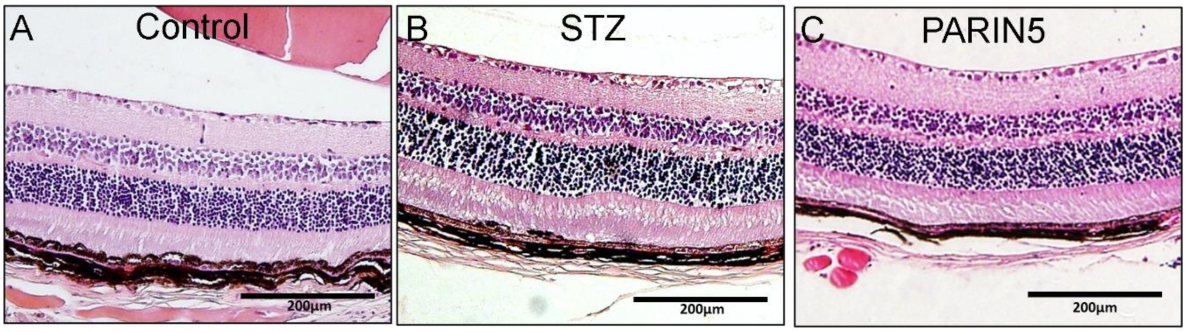

Supplementary Figure S1. Normal Retinal structure in STZ and STZ-PARIN5 treated mice: Representative hematoxylin-eosin retinal histology staining of 5 weeks STZ-induced diabetic mice retina compared to control and STZ-treated mice. Histological evaluation revealed no gross changes in the retinal layers, indicating normal retinal structures, and the absence of edema in (A) Control (N=2-3), (B) STZ (N=3-4), and (C) PAIRN5 STZ (N=3) treated mice. Scale bars 200µm. The lack of structural changes (as indicated by the H&E histology) in the relatively early diabetes stages (5 weeks following induction) suggests that the thrombin activity measures are preceding the clinical pathological observations conducted by the routine accepted means. This was further supported by Spectral domain optical coherence SD-OCT tests and multicolor fundus imaging indicated normal retinal vasculature and found no evidence of edema, neovascularization, microaneurysm, and hemorrhage in the retina.

Supplementary Table S1. MIQE checklist.

**Experimental design**			
Definition of experimental and control groups	Comparison between the control group, healthy non-diabetic mice, STZ-induced diabetic mice, and PARIN5 treated mice.		
Number within each group	Control non-diabetic mice: n=8<br>STZ induced diabetic mice: n=4<br>PARIN5 treated mice: n=3		
**Sample**			
Description	Right half brain		
Microdissection or macrodissection	Macrodissection		
Processing procedure	Fresh frozen tissue		
If frozen, how and how quickly?	Snap-frozen in liquid nitrogen within 1 minute of surgery		
Sample storage condition and duration	Storage at-80ºc until processing		
**Nucleic acid extraction**			
Procedure and instrument	Homogenization using bullet blender homogenizer- BB*24B, Next Advance, NY, USA		
Name of kit	™ Total RNA Mini Kit, 7326820		
Details of DNase treatment	Aurum DNase I, 7326828		
Nucleic acid quantification	UV-Vis Spectrophotometer		
Instrument and method	Thermo Scientific™ NanoDrop, 13-400-518		
Purity (A_{260}/A_{280})	Mean 2.141, Std. Deviation 0.029, St. Error 0.007		
**Reverse transcription**			
Amount of RNA and reaction volume	1μg in 20 μI reaction		
Reverse transcriptase and concentration	MultiScribe™ Reverse Transcriptase, 4311235,<br>50 units / 20 μI reaction		
Temperature and time	10 minutes 25°C, 120 minutes 37°C, 5 minutes 85°C		
**qPCR target information**			
Gene symbol	F2R (PAR1), F10 (FX), TNF a (TNF-α), F2 (PT)		
Sequence accession number	NM_010169 (PAR1), CT010325 (FX), 10479 (TNF-α), 88380 (PT)		
**qPCR oligonucleotide**			
Primer sequences	Gene name	Direction	Sequence
	PAR1	Forward	GCCTCCATCATGCTCATGAC
		Reverse	AAAGCAGACGATGAAGATGCA
	FX	Forward	GTGGCCGGGAATGCAA
		Reverse	AACCCTTCATTGTCTTCGTTAATGA
	TNF-α	Forward	AGATCAATCGGCCCGACTATCTC
		Reverse	GTTTGGGAAGGTTGGATGTTCGT
	PT	Forward	CCGAAAGGGCAACCTAGAGC
		Reverse	GGCCCAGAACACGTCTGTG
**qPCR protocol**			
Reaction volume and amount of cDNA	50ng/10ml reaction mix		
Primer concentration	1000nM		
Complete thermocycling parameters	1 cycle of 95° C for 20 seconds<br>40 cycles of 95° C for 3 seconds<br>60° C for 30 seconds		
Manufacturer of qPCR instrument	StepOne™ Real-Time PCR System, Applied Biosystems		
**Data analysis**			
qPCR analysis program	StepOnePlus™ Software v2.3		
Normalization method	Fixed thresholds and baseline for all samples per gene		
Statistical method for results significant	P<0.05		
